# Social support and quality of life in patients with chronic liver disease: a mediating role of illness perception

**DOI:** 10.3389/fpsyg.2026.1613767

**Published:** 2026-03-18

**Authors:** Qin Xie, Tian Ren, Teng Li, Jia Liu, Wanni Yu, Xiaoling Fu, Dongmei Li, Li Liu, Mei Yang

**Affiliations:** 1 Department of Gastroenterology, Institute of Digestive Diseases of PLA, The First Affiliated Hospital (Southwest Hospital), Army Medical University (Third Military Medical University), Chongqing, China; 2 Department of Rheumatology, Chongqing Traditional Chinese Medicine Hospital, Chongqing, China

**Keywords:** chronic liver disease, illness perception, mediating effect, quality of life, social support

## Abstract

**Background:**

Chronic liver disease is a major global health threat. With the progress of disease, patients will suffer from different complications, which seriously affect their quality of life. Social support and illness perception play key roles in improving quality of life. However, there is limited research on the relationship between social support, illness perception, and quality of life.

**Methods:**

The STROBE guideline was performed in this study. 250 individuals in a Grade A hospital in China were selected by a convenience sampling approach. Data were collected by using a sociodemographic questionnaire, a Social Support Rating Scale, a Brief Illness Perception Questionnaire, and a 36-item Short Form Health Survey Questionnaire. IBM SPSS Amos 24.0 (IBM Corp.) and SPSS 26.0 (IBM Corp.) were used for data analysis.

**Results:**

The overall mean scores of social support, illness perception and quality of life were 26.538, 43.420 and 62.641, respectively. The quality of life was positively correlated with social support, while it was negatively related to illness perception. The social support was negatively correlated with illness perception. The illness perception acted as a mediator in the relationship between social support and quality of life.

**Conclusion:**

Statistically, illness perception acts as a complete mediator in the association between social support and quality of life. Healthcare professionals should focus more on understanding and addressing patients’ illness perceptions to enhance their quality of life.

## Introduction

Chronic liver disease (CLD) is a progressive deterioration of liver functions, which gradually develops into fibrosis and cirrhosis. The total number of CLD cases is estimated at 1.5 billion worldwide (Moon et al., 2020). The most common causes of CLD are nonalcoholic fatty liver disease (NAFLD, 59%), hepatitis B (HBV, 29%), hepatitis C (HCV, 9%) and alcoholic liver disease (ALD, 2%) (Cheemerla and Balakrishnan, 2021). Incredibly, 257 million people are living with chronic HBV. 71 million people, half concentrated in six countries such as India, China, Egypt, Russia, United States, and Pakistan, are infected with chronic HCV. NAFLD has a 24% estimated prevalence rate (Younossi et al., 2016). Alcohol accounts for 30% to 50% of cirrhosis-related deaths (Moon et al., 2020). As the world is experiencing an increase in obesity and alcohol consumption, NAFLD and ALD are expected to increase.

CLD, as a leading cause of human death, is a major global health threat (De Siervi et al., 2023). In 2017, CLD caused 1.32 million deaths (GBD 2017 Cirrhosis Collaborators, 2020). In the United States, the mortality rate of patients with CLD was 10.3 per 100,000 inhabitants in 2010 (Šumskienė et al., 2015). In Canada, 2,748 deaths were attributed to CLDs in 2008. In Europe, 170,000 people die each year from CLDs (Zatoński et al., 2010). In China, the overall mortality was 7.3 per 1,000 person-years (Wu et al., 2020). Nowadays, CLD is one of the most common chronic diseases in the world (Bagherniya et al., 2018; GBD 2017 Disease and Injury Incidence and Prevalence Collaborators, 2018).

With the increasing emphasis on clinical outcomes for patients with chronic diseases, quality of life (QoL) has become a key indicator in clinical management (Younossi and Guyatt, 1998). QoL, as a measurable indicator, could be evaluated by SF-36 that contains a total of eight scales. Due to high comorbidity burden including mental, physical and social challenges, QoL of patients with CLD has been demonstrated to be impaired compared with the general population (Peng et al., 2019). Even worse, nearly 20% of these patients may die in the early stages of liver failure or hepatocellular carcinoma without effective management. Thus, it is essential to make comprehensive healthcare strategies. However, with an important hindrance being the limited understanding of the liver patients’ mental and social symptoms, relatively little attention has been paid to improving the QoL for those patients with CLD in China (Grønkjær and Lauridsen, 2021; Huang et al., 2021).

Reassuringly, illness perception (IP) plays key roles in the management of other types of chronic diseases (Koontalay et al., 2024; Xu et al., 2019). Unlike traditional biomedical approaches to illnesses, IP could explore a given individual’s beliefs and representations associated with their illness, which has become a part of patients’ understanding of their disease and treatment-related actions. Those illness beliefs are conceptualized within a framework of self-regulation known as the common-sense self-regulation model (CS-SRM) that explicates the processes by which patients become aware of a health threat, navigate affective responses to the threat, formulate perceptions of the threat and potential treatment actions, create action plans for addressing the threat, and integrate continuous feedback on action plan efficacy and threat-progression (Leventhal et al., 2016). According to the model, people make sense of a health threat by developing their own cognitive and emotional representations of a health threat (Hagger et al., 2017). Importantly, it also could be evaluated by Brief Illness Perception Questionnaire (BIPQ) that is a nine-item scale providing a rapid assessment of an individual’s perceptions of illness. Each item is designed to assess one dimension of illness perception.

Additionally, a preliminary study revealed that SS is another factors affecting QoL (Shojaei et al., 2019), which has been receiving increasing attention in the management of chronic diseases. SS is defined as the support that one receives from family, friends, organizations, and other people (Helgeson, 2003), which can be measured by Social Support Rating Scale (SSRS) that contains a total of 10 items encompassing three dimensions of social support, i.e., subjective support (Sb), objective support (OS) and utilization of support (US). Sb refers to moral support such as respect, understanding, and acceptance from family members. OS refers to support from friends, family and social networks that can meet the physical, psychological and social needs of the individual. US refers to the extent to which individuals utilize and participate in social support when they experience frustration.

**Figure 1:**
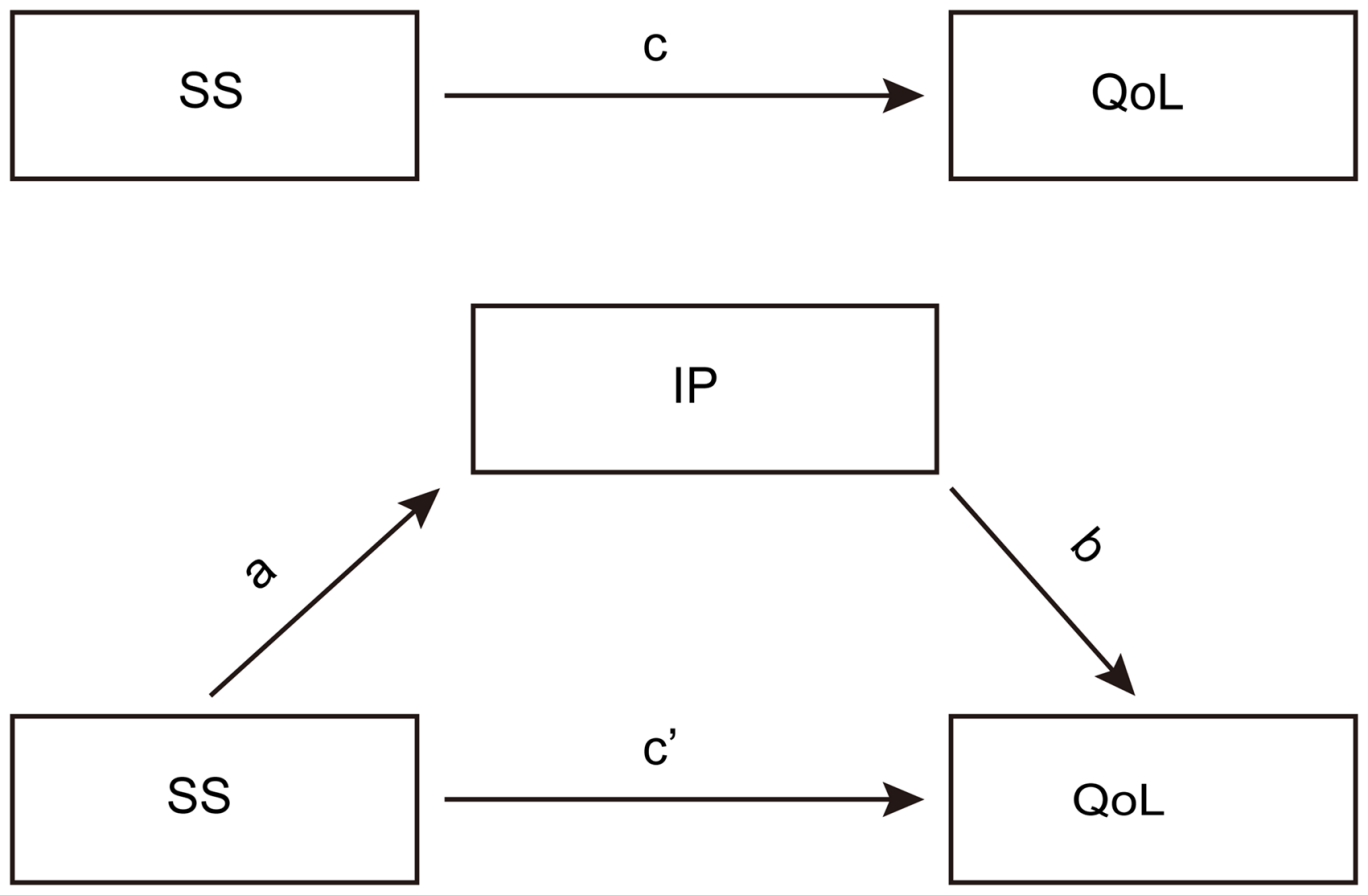
The conceptual mediation model.

While the mediational role of IP between SS and QoL is well-documented across chronic conditions (Chen et al., 2025), CLD possesses unique psychosocial characteristics that may meaningfully alter this pathway. Unlike diabetes or cardiovascular disease, CLD is often marked by prolonged asymptomatic progression, unpredictable prognosis, and significant stigma, particularly related to etiologies such as viral hepatitis or alcohol-associated liver disease, which can lead to feelings of etiology-related guilt or shame (Carol et al., 2022; Cortesi et al., 2020). These unique features suggest that the pathway between SS, IP, and QoL may operate through CLD-specific mechanisms, justifying the need for targeted investigation. The purpose of this study is to explore the relationship among IP, SS and QoL and provide comprehensive strategies for clinical management of CLD. Four hypotheses are proposed: (1) SS is correlated with QoL. (2) IP is correlated with QoL. (3) SS is correlated with IP. (4) IP mediates the relationship between SS and QoL. The theoretical model is presented in Figure 1.

## Materials and methods

### Design

A cross-sectional design with convenience sampling was conducted in this study on Chinese patients with CLD. STROBE guidelines for observational studies are followed. Although disease stage, etiology and clinical symptoms/complications are important determinants of QoL in CLD, the present study focused on psychological and social mechanisms (i.e., IP as a mediator between SS and QoL) as its primary research objective. Given the study’s focus on cognitive-appraisal and social-support pathways, and data availability limitations, these clinical variables were not included as covariates or moderators in the structural equation model (SEM). This decision allowed us to prioritize the testing of the hypothesized psychological-social framework.

### Setting and participants

**Figure 2:**
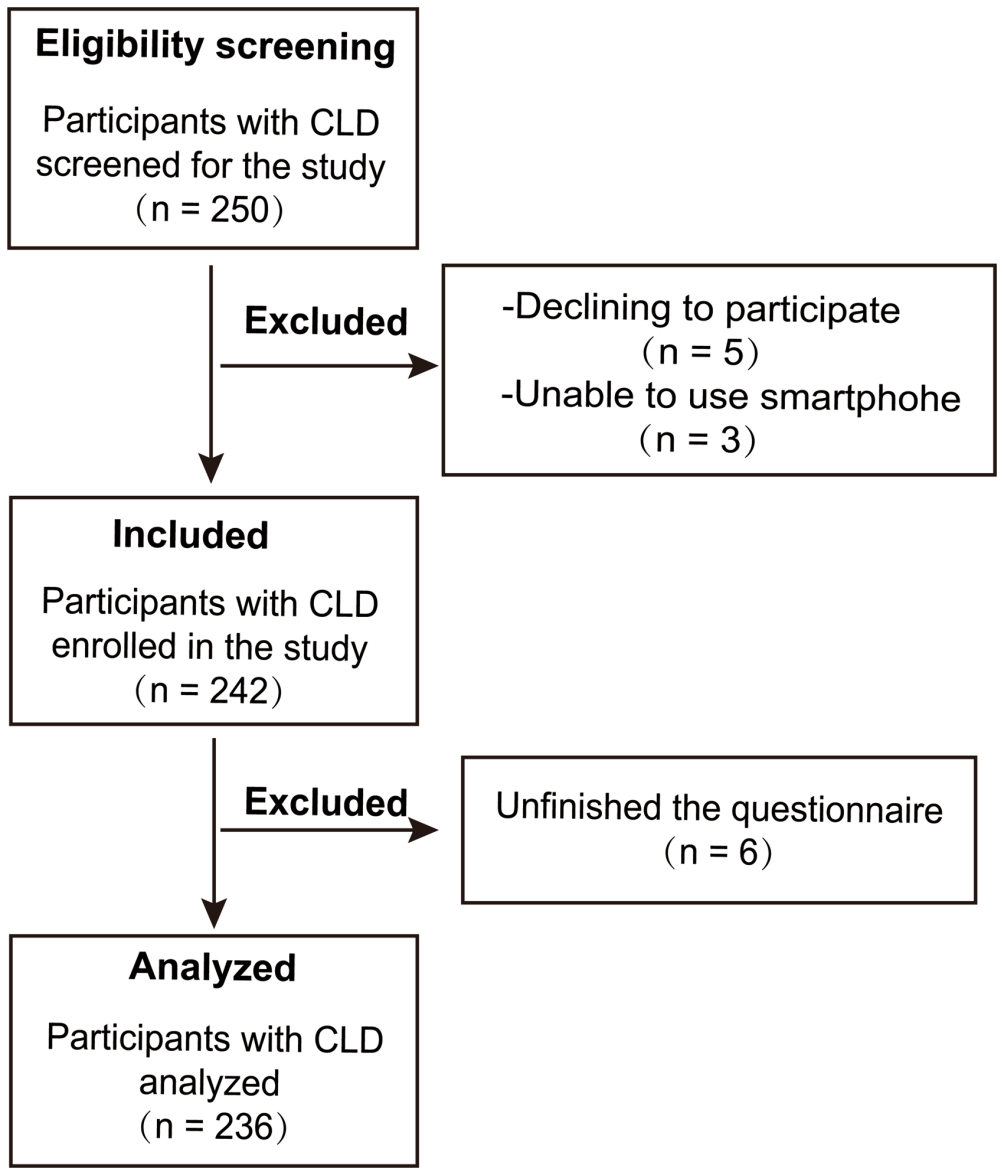
Flow diagram of the study sample.

From April to October 2023, 250 individuals with CLD were screened in a top three hospital in Chongqing City, China. After obtaining the consent of individuals, they were asked to complete a questionnaire regarding social support, illness perception and quality of life. Outpatients and inpatients were eligible for enrollment according to the following criteria: (a) individuals ≥ 18 years of age; (b) individuals diagnosed with CLD by the attending physician. Of note, their primary caregiver who lived together with individuals at least for 1 year when the questionnaire was completed by the caregiver; (c) individuals with normal speech and comprehension who were able to collaborate with researchers; (d) individuals who are voluntary and consenting. Patients were excluded from this study if they suffered from psychiatric diseases such as depression or if they had other severe diseases such as malignancy. Finally, a total of 236 participants completed questionnaires that were finally retrieved with a recovery rate of 94.4% (Figure 2). Notably, patients with a clinical diagnosis of depression were excluded for two key reasons. Clinically, severe depressive comorbidity independently and profoundly impacts IP and QoL in CLD, which could confound the observed relationships. Theoretically, our study focused on the specific cognitive-appraisal pathway of IP; pervasive negative cognitions in depression may overshadow illness-specific perceptions, obscuring the unique role of the hypothesized mediator. This exclusion allowed us to isolate the social support–illness perception–quality of life pathway without confounding from severe depressive psychopathology.

### Ethics considerations

This study received ethical approval from the Ethics Committee of the First Affiliated Hospital of Army Military Medical University (KY2024021). After obtaining informed consent from participants, the questionnaires were completed with the help of assistant researchers. Of note, these research assistants have been working for at least 5 years and have received professional training. Also, it is essential for them to have excellent communication skills. They assisted each patient in completing questionnaires by providing detailed explanations of important considerations, promptly addressing any issues encountered, and recording the results in a timely manner. The survey was performed anonymously to ensure their privacy.

### Instruments

#### Demographic characteristic form

A general sociodemographic form including gender, age, marital status (married/others), occupation (employed/unemployed/retired/others), health insurance (Medical insurance for urban employees/medical insurance for residents/Self-paying), definite diagnosis (yes/no), hospitalization frequency (below 1/2–3/above 4 times), and monthly income (under 1,000/1,000–3,000/3,000–5,000/above 5,000 RMB) was designed. Clinical information included a definite diagnosis (yes/no).

#### Social support rating scale (SSRS)

SSRS was used to evaluate the level of social support among patients with CLD. The scale was composed of three dimensions including 10 items as follows: subjective support (Sb), objective support (OS) and utilization of support (US). Questions 1–4 and 8–10 have four response options: A, B, C and D, which are worth 1–4 points, respectively. Question 5 is “Support and care received from family members and is answered on a 4-point scale ranging from 1 (none) to 4 (complete support). Question 6 is What are the sources of help and financial support you have received when you have encountered difficulties?,” and it is assessed based on the number of options, which means several sources of help. The total score of SSRS ranged from 12 to 66, with higher scores indicating better levels of social support for the subjects. The score of SSRS between 12–22 indicates poor social support, 23–44 indicates moderate social support, and 45–66 is considered to have adequate social support (Yang et al., 2023). The SSRS has been widely used to measure the level of social support in the Chinese population (Ye et al., 2022). The Cronbach’s *α* value of the SSRS in this study was 0.77.

#### Brief illness perception questionnaire (BIPQ)

The Chinese version of BIPQ was applied to assess the level of illness perception, which has been widely used in China and showed an acceptable reliability. Good construct validity of BIPQ in the population with chronic disease was reported (Broadbent et al., 2015). It comprises three components such as cognitive (Co), emotional (Em), and comprehensibility (Cm). It consists of 9 items. The first five items assess Co, specifically perceived consequences (Item 1), timeline (Item 2), personal control (Item 3), treatment control (Item 4), and the presenting symptoms or identity (Item 5). Em is assessed through concern (Item 6) and emotions (Item 8). Only one item assesses Cm (Item 7). The final question (item 9) is in an open-ended format where patients are asked to list down the three main factors that cause their illness. A higher score indicates a stronger negative perception of each dimension. The overall score ranges from a best possible score of 1 to a worst possible score of 10. The coefficients of test–retest reliability for the BIPQ ranged from 0.69 to 0.73, and the Cronbach’s *α* of the subscales ranged from 0.70 to 0.84 (Turner-Bowker et al., 2003). Cut-off points for the BIPQ total score were determined as follows: <42 indicating low experienced threat, 42–49 indicating moderate experienced threat, and ≥50 indicating high experienced threat.

#### The 36-item short form health survey questionnaire (SF-36)

The QoL was assessed by the SF-36 that operated in the Chinese population. The domains of SF-36 include eight scales: general health (GH), physical (PF) and social functioning (SF), bodily pain (BP), role physical (RP), mental health (MH), role emotional (RE) and vitality (VT). Component analyses showed that there are two distinct concepts measured by the SF-36: a physical dimension, represented by the Physical Component Summary (PCS), and a mental dimension, represented by the Mental Component Summary (MCS). Example items include “Are you satisfied with your interpersonal relationships?”, and “Do you think your life is meaningful?”, which are answered on a 5-point scale from 1 (strongly agree) to 5 (strongly disagree). The original score for each entry is converted to a standardized score ranging from 0 to 100. PSC, MCS and the total score are calculated according to the formula in the SF-36 manual. A higher score indicates a better quality of life. The Cronbach’s *α* value for the SF-36 was 0.88 (Gutteling et al., 2006; Komiya et al., 2010).

### Statistical analysis

Data were independently entered by two researchers followed by crosschecking and verification to minimize errors. Questionnaires with ≥20% missing content were considered invalid and excluded. Descriptive analyses of general demographic information and the level of QoL, SS as well as IP were performed. Continuous variables conforming to normal distribution were described by Mean ± SD (mean and standard deviation), and categorical variables were described by N (%) (frequency). Non-normal data were transformed into a normal distribution using log transformation. Categorical variables were presented as frequency (percentage). The scores of QoL, SS and IP were compared using an independent t-test between two groups and an ANOVA test between more than two groups. Pearson correlations were calculated to explore the bivariate relation among SS, IP and QoL (continuous, normal distribution). The IBM SPSS 25.0 (IBM Corp.) was used to conduct descriptive and correlational analyses. The mediating effect was analyzed by IBM SPSS Amos 24.0 (IBM Corp.) based on SEM. We adopted the Bootstrap method with 5,000 replicates to verify the hypothesis to explore the mediation effect. If the 95% confidence interval does not include 0, the mediation effect is considered significant. All *p* values are two-sided probability, and the test level is *α* = 0.05.

## Results

### Demography

In this study, the distribution of QoL was significantly different according to age (*F* = 7.478, *p* < 0.01), occupation (*F* = 8.682, *p* < 0.01), hospitalization frequency (*F* = 6.560, *p* < 0.01) and monthly income (*F* = 3.304, *p* < 0.05). There were statistically significant differences in SS based on occupation (*F* = 6.455, *p* < 0.01), diagnosis (*t* = −2.360, *p* < 0.05) and hospitalization frequency (*F* = 4.076, *p* < 0.05). The distribution of IP was significantly different according to gender (*t* = −2.009, *p* < 0.05), age (*F* = 5.282, *p* < 0.01), occupation (*F* = 4.403, *p* < 0.01), diagnosis (*t* = −2.043, *p* < 0.05) and hospitalization frequency (*F* = 14.587, *p* < 0.01) and monthly income (*F* = 4.385, *p* < 0.01). General data are shown in Table 1.

**Table 1:** Sociodemographic characteristics (*N* = 236).

Variables	*N*	Quality of life			Social support			Social support		
		M (SD)	Statistics	*p*	M (SD)	Statistics	*p*	M (SD)	Statistics	*p*
Gender										
Male	131	63.162 (16.649)	*t* = 0.533	0.594	26.777 (6.185)	*t* = 0.655	0.513	42.153 (10.442)	*t* = −2.009	0.046^*^
Female	105	61.992 (16.893)			26.238 (6.450)			45.010 (11.352)		
Age										
≤40	56	70.625 (15.555)	*F* = 7.478	0.000^**^	27.036 (7.333)	*F* = 0.547	0.650	38.643 (10.116)	*F* = 5.282	0.002^**^
41–50	49	60.094 (18.148)			27.163 (6.401)			44.612 (11.105)		
51–60	78	62.593 (15.359)			25.910 (5.880)			44.269 (10.481)		
≥61	53	56.632 (15.634)			26.359 (5.657)			46.132 (10.965)		
Marital status										
Married	187	62.099 (16.758)	*t* = 0.973	0.332	26.670 (6.226)	*t* = −0.340	0.734	43.834 (10.741)	*t* = −1.128	0.260
Others	49	64.712 (15.618)			26.265 (6.617)			41.857 (11.587)		
Occupation										
Employment	69	69.891 (15.905)	*F* = 8.682	0.000^**^	29.174 (5.933)	*F* = 6.455	0.000^**^	40.188 (9.941)	*F* = 4.403	0.005^**^
Unemployment	35	54.000 (15.132)			25.000 (6.226)			47.971 (9.931)		
Retirement	55	60.093 (16.105)			26.146 (5.592)			44.509 (11.164)		
Others	77	61.893 (16.287)			25.156 (6.487)			43.481 (11.320)		
Source of insurance										
Government	120	60.157 (16.596)	*F* = 2.780	0.064	25.942 (6.270)	*F* = 2.371	0.096	44.342 (10.729)	*F* = 0.945	0.390
Company	98	65.418 (17.134)			27.551 (6.300)			42.296 (10.896)		
Self-paying	18	64.083 (12.999)			25.000 (5.951)			43.444 (12.370)		
Diagnosis										
Yes	197	61.865 (16.628)	*t* = 1.608	0.109	26.965 (6.384)	*t* = −2.360	0.019^*^	44.066 (10.829)	*t* = −2.043	0.042^*^
No	39	66.564 (16.921)			24.385 (5.408)			40.180 (10.976)		
Hospitalization frequency										
≤1	112	66.303 (17.801)	*F* = 6.560	0.002^**^	26.125 (6.450)	*F* = 4.076	0.018^*^	40.848 (10.642)	*F* = 14.587	0.000^**^
2 ~ 3	47	62.266 (14.482)			28.830 (6.475)			41.000 (11.617)		
≥4	77	57.546 (15.159)			25.740 (5.683)			48.649 (8.998)		
Monthly income (RMB)										
<1,000	51	58.875 (16.135)	*F* = 3.304	0.021*	25.824 (6.674)	*F* = 1.878	0.134	47.549 (10.435)	*F* = 4.385	0.005^**^
1,000 ~ 3,000	64	60.828 (16.642)			25.436 (5.751)			44.000 (11.050)		
3,000 ~ 5,000	71	62.803 (15.072)			27.056 (6.110)			42.183 (10.739)		
>5,000	50	68.575 (18.478)			27.940 (6.656)			40.240 (10.438)		

SD, standard deviation; *p < 0.05, **p < 0.01.

### Bivariate analysis

The overall mean scores of SS, IP and QoL were 26.538 ± 6.296, 43.420 ± 10.926 and 62.641 ± 16.748, respectively (Table 2). A Pearson correlation coefficient was calculated to explore the bivariate relation among SS, IP and QoL. The results demonstrated that QoL was positively correlated with SS (*r* = 0.286, *p* < 0.01). The findings also indicated a significant negative relationship between QoL and IP (*r* = −0.623, *p* < 0.01). A significant negative correlation was found between SS and IP (*r* = −0.210, *p* < 0.01). As a result, these findings support the first three hypotheses. The correlation between SS, IP, and QoL is shown in the Table 2.

**Table 2:** Bivariate correlation among variables (*N* = 236).

Variables	Mean (SD)	1	2	3	4	5	6	7	8	9	10	11
1. SS	26.538 (6.296)	1										
2. Sb	8.540 (2.401)	0.596**	1									
3. OS	10.830 (4.457)	0.835**	0.176**	1								
4. US	7.170 (2.027)	0.563**	0.282**	0.188**	1							
5. IP	43.420 (10.926)	−0.210**	−0.183**	−0.110	−0.194**	1						
6. Co	25.420 (8.562)	−0.198**	−0.175**	−0.094	−0.203**	0.908**	1					
7. Em	13.470 (4.757)	−0.016	−0.029	0.015	−0.047	0.561**	0.271**	1				
8. Cm	4.530 (3.031)	−0.173**	0.120	−0.155*	−0.054	0.159*	0.022	−0.311**	1			
9. QoL	62.641 (16.748)	0.286**	0.162*	0.230**	0.192**	−0.623**	−0.565**	−0.344**	−0.110	1		
10. MCS	64.481 (17.829)	0.271**	0.187**	0.189**	0.203**	−0.553**	−0.473**	−0.343**	−0.118	0.919**	1	
11. PCS	60.839 (18.497)	0.257**	0.114	0.233**	0.152**	−0.596**	−0.568**	−0.292**	−0.083	0.925**	0.700**	1

Sb, Subjective support; OS, Objective support; US, Utilization of support; Co, Cognition; Em, Emotion; Cm, comprehensibility; MCS, Mental component summary; PCS, Physical component summary; SD, standard deviations.

*p < 0.05, **p < 0.01.

### Model test

**Figure 3:**
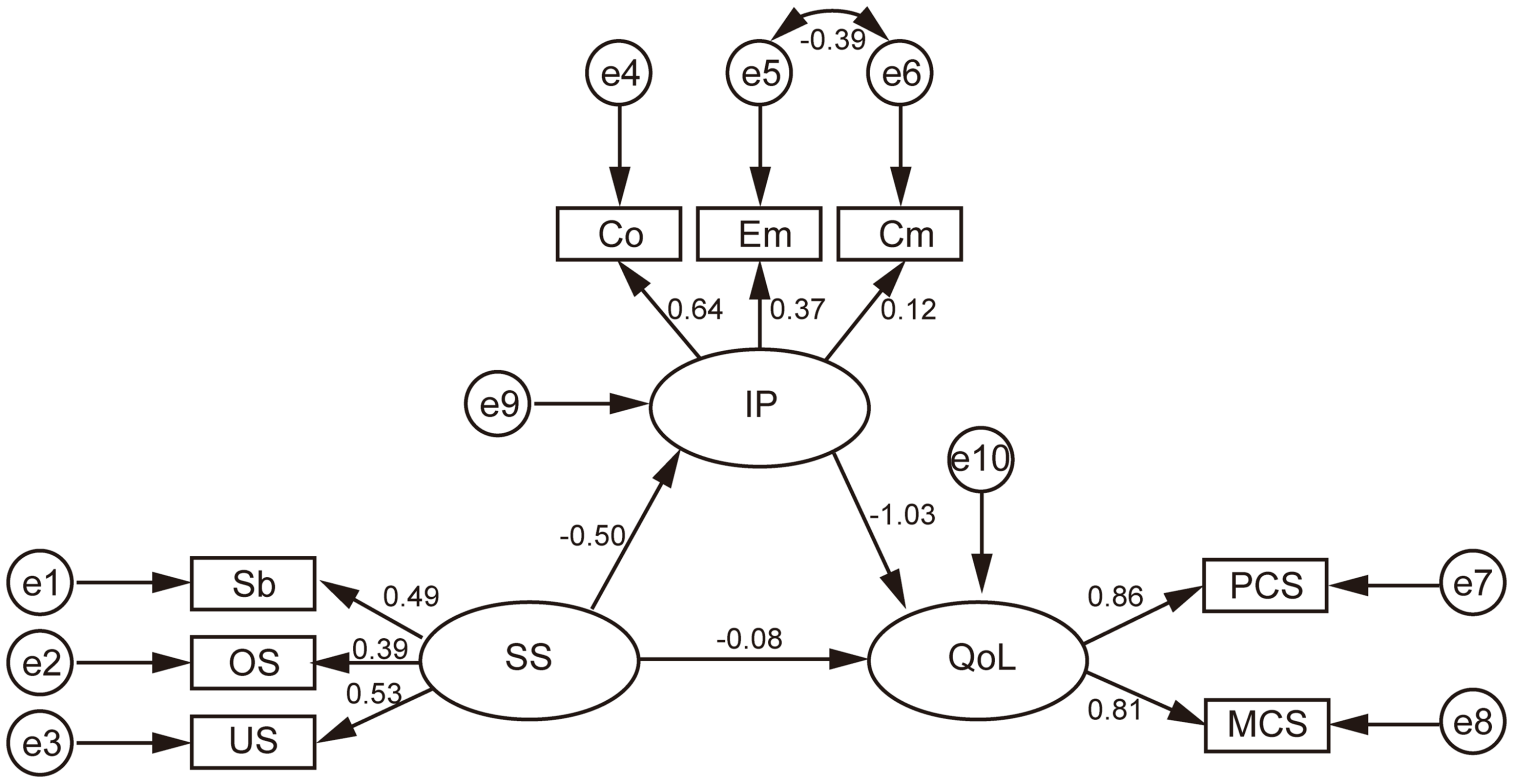
Final model with standardized estimates.

To investigate the mediating effect, the structural equation model and observation variables were used. The final model with standardized estimates was shown in Figure 3. The model has good statistical evidence for all indexes (*χ*^2^/df = 1.735, RMSEA = 0.056, CFI = 0.968, IFI = 0.969, GFI = 0.972, SRMR = 0.052, NFI = 0.930). The bootstrap operation of the process was performed with 5,000 bootstrap samples. As shown in Table 3, the total effect (path c) of SS on QoL was 0.428 (*p* < 0.01). The significant coefficients of path a and path b were −0.499 [*p* < 0.01, 95% CI (−0.804, −0.210)] and −1.025 [*p* < 0.01, 95% CI (−1.998, −0.7650)], respectively. In addition, the point estimate of the indirect effect (a*b) between SS and QoL *via* IP was 0.511 [*p* < 0.01, 95% CI (0.197, 1.796)] and that the 95% CI interval did not include 0, suggesting that there was a mediating effect. Moreover, the direct effect of SS on QoL was −0.084 [*p* = 0.635, 95% CI (−1.117, 0.257)], suggesting that there was no direct effect between SS and QoL. Statistically, IP acts as a complete mediator in the association between SS and QoL in this population. The results of SEM support our fourth hypothesis.

**Table 3:** The breakdown table of total, direct and indirect effects for the mediating model.

Model pathways	Symbol	Effect	B	95% CI		*p*
				LL	UL	
SS—IP—QoL	a*b	Indirect effect	0.511	0.197	1.796	0.000
SS—IP	a		−0.499	−0.804	−0.210	0.001
IP—QoL	b		−1.025	−1.998	−0.765	0.000
SS—QoL	c’	Direct effect	−0.084	−1.117	0.257	0.635
SS—QoL	c	Total effect	0.428	0.431	1.081	0.000

95% CI LL and 95% CI UL refers to lower limit and upper limit of 95% confidence interval of each effect estimated by deviation corrected percentile bootstrap method, respectively.

## Discussion

Our research revealed that the scores of PCS and MCS were 60.839 and 64.481, respectively. These scores are higher than those reported in earlier studies in 2013 (Gao et al., 2013), indicating that QoL of patients with CLD in China has been improved. The reasons for improvement may be as follows: (1) Over the past ten years, with the development of the economy, there has been increased focus on nursing interventions and patients’ self-management in China. Meanwhile, the whole society works together to raise awareness and alleviate the stigma associated with CLD, significantly contributing to a decrease in mortality rates (Sun et al., 2019). This shift may have helped reduce psychological stress among patients. (2) QoL is known to vary based on the stage of the disease. As CLD progresses from a non-cirrhotic to an advanced cirrhotic stage, a decline in QoL is observed (Gao et al., 2012). At the advanced stage of CLD, patients suffered from fatigue, loss of self-esteem, inability to function at work, anxiety, depression, and other emotional problems that severely diminish their QoL and well-being. To delay the progression of the disease, it is imperative for healthcare professionals and patients or their caregivers to enhance the management of disease.

Social support showed no direct effect on QoL, with statistical full mediation by IP. In contrast, patients with diabetes or hypertension have more tangible symptom markers (e.g., blood glucose, blood pressure) that allow for direct QoL impacts from SS independent of IP. CLD patients often lack the resources to understand their illness or cope with its consequences, making IP the sole pathway linking SS to QoL, likely due to CLD’s unique stigma, complex and unpredictable disease course and etiology-related guilt amplifying patients’ dependence on SS to form coherent illness appraisals. For interventions, targeting IP may be more impactful than generic SS. Prior researches illustrated that self-management behaviors are influenced by IP (Xiong et al., 2023) and better clinical outcomes can be achieved by addressing IP. IP is emotional and cognitive representations of health threats, and reflect patients’ own beliefs about their illness. Higher scores indicate a higher perceived threat regarding the illness (Kuiper et al., 2022). In our study, the average score of IP was 43.420 points. Based on a cut-off score of ≥ 42 points (Kuiper et al., 2022), patients with CLD may be considered to have at least a moderate threatening view of their illness. Person and family-centered care (PFCC) could help patients better manage complex chronic conditions and have reduced anxiety and stress (Osborn and Squires, 2012). Also, cognitive-behavioral therapy (CBT), as an evidence-based psychological intervention for treating anxiety disorders, is widely used to help patients develop more positive and adaptive perceptions (Preston et al., 2011). Of note, the coping style has been identified as a mediator between IP and QoL in other chronic disease (Tu et al., 2022). Further research is needed to determine whether IP affects QoL by coping style among patients with CLD. These findings collectively provide additional insights for enhancing the management of disease by addressing IP.

The present findings extend the general chronic illness literature by validating the mediational model in a population with unique psychosocial challenges. Specifically, the stigma and etiology-related guilt inherent to CLD may amplify the role of IP. Patients with stronger SS may be better able to reframe negative IP (e.g., reducing guilt or perceived stigma), thereby protecting QoL—a mechanism that may be more pronounced in CLD than in conditions with less stigma. Additionally, the asymptomatic yet potentially life-threatening nature of CLD may make patients’ cognitive appraisals particularly salient in translating SS into adaptive outcomes. Thus, this study refines existing models by demonstrating their applicability and contextual relevance to CLD, providing a CLD-specific framework for understanding psychosocial adaptation and informing targeted interventions.

However, there are certain limitations to this study. First, the research’s cross-sectional design makes it impossible to establish causal relationships. The statistical full mediating effect of IP only reflects a correlational pattern in the study sample at a single time point, rather than a definitive causal pathway. Reverse causality cannot be excluded. Additionally, unmeasured confounding variables (e.g., disease severity, duration of CLD, or prior psychological interventions) may affect the observed associations. Future longitudinal studies or intervention studies are required to verify the potential causal direction of these relationships. Second, a notable limitation of this study is the omission of key clinical factors known to influence QoL in CLD, including disease stage (e.g., fibrosis, compensated/decompensated cirrhosis), etiology (e.g., NAFLD, HBV, HCV), and the presence of major symptoms or complications (e.g., ascites, hepatic encephalopathy). These clinical indicators may act as confounders or moderators of the observed relationships between SS, IP, and QoL. The lack of clinical data thus limits the generalizability of our findings. Future research should integrate objective clinical measures (e.g., disease stage, etiology, Child-Pugh score, laboratory markers, and complication status) into mediation models to clarify whether the mediating role of IP varies by disease stage or etiology in CLD. Including these clinical factors as covariates or moderators would allow for a more nuanced understanding of how SS and IP operate across different clinical profiles, and improve the clinical relevance and predictive utility of the model. Third, convenience sampling from a single tertiary hospital may introduce selection bias and limit external validity, restricting generalizability to other CLD populations and settings. Excluding patients with depression may have systematically biased the sample toward better psychosocial functioning. Given the high prevalence of depression in CLD and its strong association with negative IP and poor QoL, this exclusion may have attenuated the strength of the observed relationships, limiting applicability to patients with comorbid depression. Therefore, reducing biases should be focused on by evaluating and controlling the representativeness of a sample in further research. Also, the diversity of samples should be increased through distributing questionnaires at different times and locations to achieve an appropriate cross-section of the target population. Additional longitudinal studies are necessary for a more accurate assessment.

## Conclusion

In conclusion, the achievements of this research illustrate that social support and illness perception were considerably related with the quality of life in Chinese patients with CLD. Statistically, illness perception acts as a complete mediator in the association between social support and quality of life. As such, healthcare professionals should focus more on understanding and addressing patients’ illness perceptions to enhance their quality of life.

## Data Availability

The raw data supporting the conclusions of this article will be made available by the authors, without undue reservation.

## Glossary

CLD: chronic liver disease
NAFLD: nonalcoholic fatty liver disease
HBV: hepatitis B
HCV: hepatitis C
ALD: alcoholic liver disease
QoL: quality of life
SS: social support
IP: illness perception
CS-SRM: common-sense self-regulation model
SSRS: Social Support Rating Scale
BIPQ: Brief Illness Perception Questionnaire
SF-36: The 36-Item Short Form Health Survey Questionnaire
Sb: subjective support
OS: objective support
US: utilization of support
Co: cognition
Em: emotion
Cm: comprehensibility
MCS: Mental Component Summary
PCS: Physical Component Summary
SD: standard deviations
PFCC: person- and family-centered care
CBT: Cognitive-behavioral therapy
